# Navigating Life Transitions as a Strategy for Preventing Late-Life Suicide

**DOI:** 10.1093/geroni/igaf122.1212

**Published:** 2025-12-31

**Authors:** Catherine Ju, Amy Fiske

## Abstract

Older adults have higher rates of suicide than the general population. Life transitions and changes unique to aging may contribute to this increased risk of suicide. This symposium will feature research examining the identification of suicide risk factors and prevention of suicide in older adults, with a particular focus on the role of changes in functioning and health. First, Catherine Ju will present research investigating how strategies older adults might use to compensate for functional impairment (e.g., seeking help) might play a role in risk for suicidal ideation among people who highly value their own autonomy. Second, Dr. Kimberly Van Orden will present results of a clinical trial testing whether an intervention addressing barriers to social connection (produced by functional impairment and health problems) results in reductions in suicidal ideation as measured via ecological momentary assessment. Last, Dr. Briana Mezuk will describe research employing large language models to identify and characterize the structure of life transitions of older suicide decedents using the National Violent Death Reporting System. Dr. Amy Fiske will serve as discussant.

